# Why choice of metric matters in public health analyses: a case study of the attribution of credit for the decline in coronary heart disease mortality in the US and other populations

**DOI:** 10.1186/1471-2458-12-88

**Published:** 2012-01-28

**Authors:** Hebe N Gouda, Julia Critchley, John Powles, Simon Capewell

**Affiliations:** 1 Institute of Public Health, Forvie Site, Robinson Way, University of Cambridge, Cambridge CB2 1SP, UK; 2 School of Population Health, University of Queensland, Brisbane, Australia; 3 Institute of Health and Society, Newcastle University, Leech Building, The Medical School, Newcastle-upon-Tyne NE2 4HH, UK; 4 Division of Population Health Sciences and Education, St George's University of London, Cranmer Terrace, London SW17 0RE, UK; 5 Division of Public Health, University of Liverpool, Whelan Building, The Quadrangle, Brownlow Hill, Liverpool L69 3 GB, UK

**Keywords:** Comparative Effectiveness Research, Policy analysis, Determinants of Mortality, Epidemiologic Methods, Coronary Heart Disease

## Abstract

**Background:**

Reasons for the widespread declines in coronary heart disease (CHD) mortality in high income countries are controversial. Here we explore how the type of metric chosen for the analyses of these declines affects the answer obtained.

**Methods:**

The analyses we reviewed were performed using IMPACT, a large Excel based model of the determinants of temporal change in mortality from CHD. Assessments of the decline in CHD mortality in the USA between 1980 and 2000 served as the central case study.

**Results:**

Analyses based in the metric of number of deaths prevented attributed about half the decline to treatments (including preventive medications) and half to favourable shifts in risk factors. However, when mortality change was expressed in the metric of life-years-gained, the share attributed to risk factor change rose to 65%. This happened because risk factor changes were modelled as slowing disease progression, such that the hypothetical deaths averted resulted in longer average remaining lifetimes gained than the deaths averted by better treatments. This result was robust to a range of plausible assumptions on the relative effect sizes of changes in treatments and risk factors.

**Conclusions:**

Time-based metrics (such as life years) are generally preferable because they direct attention to the changes in the natural history of disease that are produced by changes in key health determinants. The life-years attached to each death averted will also weight deaths in a way that better reflects social preferences.

## Background

Public health policy is powerfully influenced by assumptions about past successes. In the last decades of the 20th century, most high income countries experienced substantial declines in premature deaths from major vascular diseases. Interpretation of the reasons for these declines remains relevant to contemporary deliberations on public health policy.

The coronary heart disease model, IMPACT, which was first developed by Capewell and colleagues in the late 1990s has been used to estimate the relative contributions of risk factor changes and clinical treatments to observed declines in coronary heart disease (CHD) mortality. As initially developed, IMPACT expressed mortality declines in the metric of 'deaths prevented or postponed' (DPP). Assessments, using DPP, have been published of the relative contribution of risk factor changes and better treatment to CHD declines in Scotland [1], England and Wales [2], Ireland [3], the USA [4], Poland (unpublished), New Zealand [5], Finland [6] China (Beijing) [7] and elsewhere.

**Figure 1:**
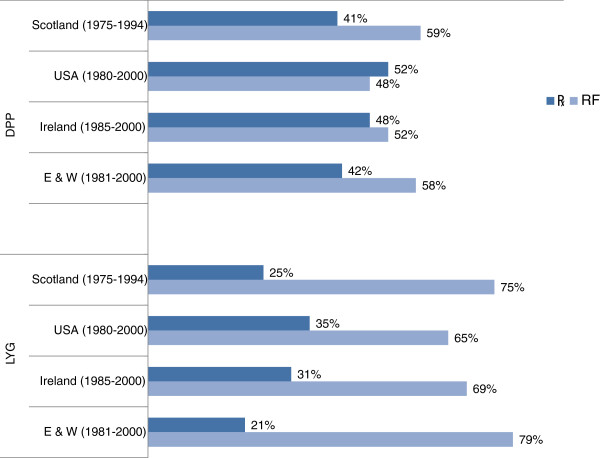
**Results of pairs of studies using IMPACT**: estimates for percentage contributions of better treatments and favorable risk factor changes to observed reductions in the risk of death from Coronary Heart Disease comparing the estimated contributions to deaths prevented or postponed (DPP, top) with those estimating contributions to life years gained (LYG, bottom).

For some of these populations Capewell *et al *have extended their analyses by expressing the mortality decline in the metric of life years gained (LYG): England and Wales [8], Scotland [9], Ireland [10] and the US [11]. A comparison of the pairs of results in these 4 countries shows that a much larger share of the credit was attributed to risk factor changes when analyses were based in LYG (Figure 1).

### Pairs of studies: DPP versus LYG

IMPACT is a very large model implemented in Excel. It uses data on age and sex specific levels of up to 6 population-based risk factors as well as nine specific treatments for nine disease states, all stratified by age and sex.

Results of four pairs of studies using the IMPACT model are shown in Figure 1. The studies using DPP attributed between one half and two thirds of the gains from 1970 to 2000 to favourable shifts in risk factor levels. However, the corresponding analyses using LYG have attributed much more of the gains (up to 79%) to RF changes.

Despite the sensitivity of results to the choice of metric, there has been little published discussion of the respective merits of models based in these alternative metrics. Here we explore why the results obtained in these analyses turn out to be so sensitive to the choice of metric. We also assess the robustness of the associated assumptions. Our review concentrates mainly on the decline in the population risks of CHD death in the USA between 1980 and 2000 [4,11] and addresses two questions: how do the differences in the attribution of credit come about? And do the extra assumptions required for the LYG analyses make them less robust? In the discussion we reflect on the wider implications of this case study.

## Methods and Results

### How does the difference in attributed credit come about?

Within IMPACT, reduced population risks of CHD death derive from two sources:

1 Better treatment: By definition this only applies to those patients who come to clinical attention, either because of the onset of clinical CHD or the detection of elevated risk factors. Treatment benefits derive either from reduced case fatality due to clinical management of an acute or chronic episode of CHD or from the therapeutic (pharmacological) reduction in elevated blood pressure or blood cholesterol levels (statin-induced LDL-C lowering for example). The gains from preventive treatments (such as statins) are shown distinct from risk factor improvement gains, (such as following reduction in population levels of cholesterol). To gain credit for a 'death prevented or postponed' an advance in treatment has to result in survival for at least 12 months longer than expected under baseline treatment. For the corresponding analyses using LYG, each death averted by advances in treatment is assigned an expected remaining life time (derivation discussed further below). The sum of these provides the LYG from treatment advances. When calculating LYG, the expected remaining lifetime is estimated using mortality rates observed in those surviving beyond the first thirty days after the onset of clinical CHD.

**Figure 2:**
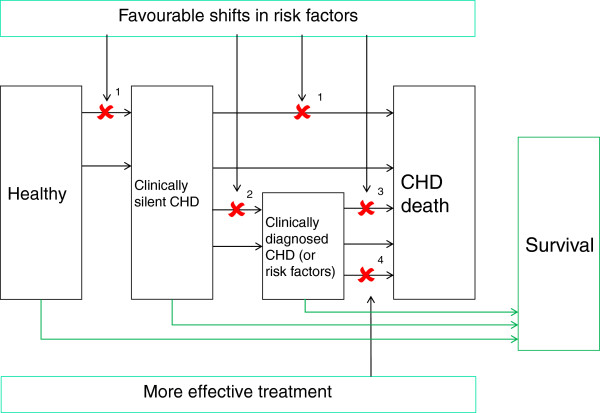
IMPACT, schematic representation of transition probabilities reduced by favorable changes in risk factors and better treatments*.

2 Risk factor changes: Shifts to more favourable risk factor levels are modelled to reduce progression through the natural history (Figure 2). This increases the person time spent in states with lower risks of fatal CHD - which, in turn, reduces deaths (countable as DPP as outlined above). For each death averted, an expected remaining lifetime is assigned, based on the stage of the natural history to which the individual has progressed. The sum provides the LYG [12-15].

* This is a simplification of the actual model, in which transitions between a larger range of states are modelled

### Key for Figure 2

Weights:

1- General US life expectancy

2- Mid-way between general US life expectancy and survival rate for post-AMI patient group

3- Equivalent to the survival rate for the post-AMI patient group

4- Patient group specific survival rates

The overall findings from these analyses are summarized in Figure 1 and Table 1. The last column in Table 1 shows that each death averted by better treatment is estimated to generate about half as many extra years lived as is each death averted by favorable shifts in risk factors.

**Table 1:** Results of pairs of studies using the IMPACT model: for each of 4 countries one study was performed using Deaths Prevented or Postponed (DPPs) and one using Life Years Gained (LYG).

	DPPs (% share)	LYG (% share)	LYG per DPP
USA 1980-2000 [4,11]			

Better treatment	159,300 (52%)	1,092,300 (35%)	6.9

Favourable risk factor changes	149,600 (48%)	2,055,600 (65%)	13.7

Ireland 1985-2000 [3,10]			

Better treatment	1640 (47.5%)	14,505 (31%)	8.8

Favourable risk factor changes	1810 (52.5%)	32,705 (69%)	18

England and Wales 1981-2000 [2,8]			

Better treatment	25,765 (42%)	194,145 (21%)	7.5

Favourable risk factor changes	35,830 (58%)	731,270 (79%)	20.4

Scotland 1975-1994 [1,9]			

Better treatment	1862 (41%)	12025 (25%)	6.5

Favourable risk factor changes	2674 (59%)	35991 (75%)	13.5

Data are the population totals for DPP and LYG (% share) and the ratios of LYG to DPP

*DPP *= Deaths Prevented or Postponed *LYG *= Life Years Gained

**Figure 3:**
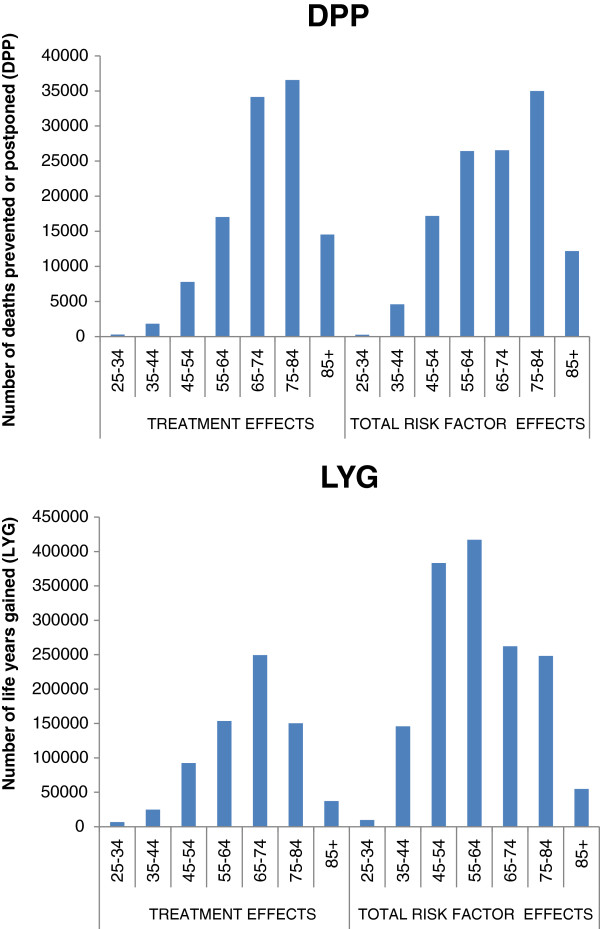
**IMPACT results for US men in 2000 relative to 1980**: Deaths prevented or postponed (DPP) and life years gained (LYG) attributed to better treatment and to risk factor changes by age (for DPP) and age of averted onset (LYG)*.

In Figure 3 the DPP and LYG are plotted against the age of the averted death. Not surprisingly, when each death averted is weighted by its LYG, changes in population disease experience at earlier ages contribute more to the aggregated benefit. Deaths averted before age 65 by favourable shifts in risk factors contribute 15.9% of the total DPPs but 36.2% of total LYGs.

* All LYG are attributed to the age of averted death, not to the age at which the life years would have been lived

### Where do the gained years come from?

The treatment arm of the US IMPACT model estimates patients' median survival using population-based data from unselected cohorts of MediCare patients following their hospital admission for acute myocardial infarction, heart failure, or revascularization. Additional age-specific median survival data for unstable angina patients were obtained from a large retrospective cohort study of unselected patients in the United Kingdom [16-18], since MediCare only included US patients aged over 65.

The risk factor arm of the model estimates survival using the life expectancy of the general US population. Favourable shifts in risk factors both prevent and postpone (clinical) onset of CHD. It is expected, therefore, that some will live with asymptomatic CHD until their first CHD event - which results in death. For this group the post-AMI (excluding heart failure) rates are employed (the maximum estimate is set at halfway between the life expectancy of post-AMI patients and the general population and the minimum estimate is set at the life expectancy of post-AMI patients including those with heart failure). A portion of the CHD patients will also be expected to have their deaths postponed due to beneficial risk factor changes. This group is assumed to have approximately the same life expectancy as patients surviving after an uncomplicated AMI (Table 2). The main survival functions employed are illustrated in Table 2.

**Table 2:** Life expectancies (e_x_) assigned to US men and women in 2000, comparing those who have not progressed to clinical CHD ('healthy') with those who have survived an acute myocardial infarction without the onset of heart failure and those who progress to heart failure

Age (x)	Life expectancy at exact age (e_x_)		
	
	Healthy population^1^	Those who have survived an AMI by 30 days (excluding those with HF*)^2^	Those who have survived an AMI by 30 days but remain in HF* ^3^
MALES			

30	45.9	28.0	14.8

40	36.7	28.0	7.4

50	27.9	14.6	5.2

60	19.9	9.7	3.6

70	13.0	6.8	1.8

80	7.6	4.1	1.5

90	4.1	2.6	1.2

100+	2.4		

FEMALES			

30	50.6	28.0	14.8

40	41.0	28.0	7.4

50	31.8	14.6	5.2

60	23.1	9.7	3.6

70	15.5	6.8	1.8

80	9.1	4.1	1.5

90	4.8	2.6	1.2

100+	2.7		

* Heart failure

^1 ^Source: US Government Actuary's Office [17]

^2 ^Source: Based upon median survival estimated from case fatality data obtained from MediCare [4]

^3 ^Source: Based upon median survival estimated from case fatality data obtained from [4]

### How robust are the model extensions required for the LYG metric?

The US IMPACT model uses five main data sources to estimate survival and life expectancy (case-fatality rates for post-AMI, heart failure, revascularization and unstable angina patient groups and the general US life expectancy) which are subsequently used to apply weights to each CHD death prevented or postponed for both the treatment and the risk factor arms of the model. These weights are adjusted for different contexts and for the specific groups of people who benefit from clinical treatments or risk factor changes according to informed assumptions (listed in Table 3).

**Table 3:** Survival functions assigned to specified health states: sources, limitations and strengths

	Data used	Data source	Limitations of assumption	Strengths of assumption
**Treatments**	CHD patients. Heart failure (HF) in the community is assumed to result in one third the fatality as HF in hospital and hypertension life expectancy is 80% that of US general population	MediCare [4]	May overestimate the life years lost due to deaths caused by HF in the community and hypertension.	May overestimate the impact of treatments preventing and postponing deaths due to HF in the community and hypertension therefore making the estimated relative contribution of risk factors conservative.

**Risk Factors**	CHD patients. Median survival was assumed to be that of the post-AMI patients	MediCare [4]	May underestimate the benefit of reductions in risk factor prevalence	Provides a conservative estimate
	
	Healthy population. survival was assumed to be that of general US population	US Bureau of the Census [17]	May both underestimate and overestimate the benefit or harms in terms of survival for each of the changes in risk factor prevalence	Avoids methodological issues of non-additivity and double-counting
	
	Asymptomatic CHD. Survival was estimated as half-way between that of post-AMI patient and General US life expectancy	MediCare and US Bureau of the Census [4,17]	Arbitrary and may underestimate the benefit of reductions in risk factor prevalence	Provides a conservative estimate

For all healthy people who never experience a CHD event, the model uses a universal weight, the general US life expectancy (weight 1 in Figure 2). There are two potential problems with this. First, the general US population is made up of people who are both at risk of a CHD death and those that are not at risk. The impact of decreasing one's exposure to a risk factor or risk factors, however, might be expected to improve one's life expectancy above that of the general population. The assumption used here in the IMPACT model, therefore, is likely to lead to conservative estimates of the gains due to risk factor changes amongst the healthy population.

Second, a universal life expectancy applied to deaths averted amongst the 'healthy' populations due to risk factors changes means that the specific benefits gained from each risk factor change are lost. There are two reasons why this one weight is employed however. First the IMPACT model calculates the number of DPPs according to specific risk profiles and the prevalence of each risk factor. Any attempt to account for these differences again would potentially double count the impact.

A separate but related issue arises from risk factors being correlated [19] so the impact of changes in individual risk factors is not additive. Thus, employing a common life expectancy for all those who avert a CHD death due to risk factor changes avoids the methodological problems that arise from non-additivity.

For the group of people with asymptomatic and/or undiagnosed CHD, the model uses a weight that is mid-way between the general US life expectancy and the survival rates of post-AMI patients (weight 2 in Figure 2). Furthermore, people diagnosed with CHD may gain survival time either from better treatment or from favourable risk factor changes. This group are given a survival weight equivalent to that of the post-AMI patient group (weight 3 in Figure 2).

These assumptions suggest that these groups of people have a small advantage in terms of survival and, again, probably provide a conservative assessment of the impact of risk factors on these groups of people.

Lastly, patient group specific survival rates are used to weight the death prevented or postponed from a treatment (weight 4 in Figure 2). Weight 4 is based on case fatality data for most of the patient groups included in the model. Two main groups, however, community heart failure and hypertension, lack sufficient data to inform on survival. To estimate survival for these two groups the model uses the assumptions that those experiencing heart failure in the community have one third of the case fatality of those who suffer heart failure in the hospital and those with hypertension will have 20% less life expectancy than the average life expectancy. Little literature exists to inform these estimates and these assumptions may slightly overestimate the number of years lost due to these conditions [20].

To answer the question posed above, therefore, all groups of people that may have avoided or postponed a CHD death have been accounted for in the US IMPACT model and the assumptions used to extend this analysis from the event-based metric DPP to the time-based metric LYG may at times seem speculative and even arbitrary, but the estimates are typically reasonable and often conservative. In other words, the model is likely to underestimate gains in life years from favourable changes in risk factors.

## Discussion

Changes in disease determinants gain policy relevance from their effects on the population's burden of disease. This applies to both risk factor changes and to therapies. We have shown how these effects are better captured when expressed in a time-based metric such as life years gained (LYG).

Extending the IMPACT model to enable outputs in LYG required additional assumptions but these turned out to be relatively robust and unlikely to seriously offset the wider advantages of the LYG model.

Although results from analyses based in LYG differ importantly in their policy implications from analyses based in DPP, it is striking that there has been little discussion of the respective merits of public health models employing these two alternative metrics. Given that public health modelling influences public health policy, it is important to 'get it right'. Criteria are therefore needed for the critical appraisal of such models. From our demonstration that choice of metric can be highly consequential in such models it follows that reports of modelling exercises should explicitly justify their choice of metric.

Time-based metrics will usually be better because:

a They direct attention to changes in the natural history of the disease produced by the change in health determinants This aids understanding of 'what is going on' in the population's health experience;

b Life years gained relate more closely to the social value of the changes in population health. Deaths, considered as instantaneous events, are weightless. It is the life foregone that matters.

Some limitations of this investigation should be noted. Furthermore, the many assumptions and limitations of the US IMPACT model have previously been noted [4,11]. In this review of the IMPACT model we have therefore focussed mainly on the assumptions involved in estimating LYG due to CHD treatments and risk factor changes, and not on those involved in estimating the underlying deaths postponed or prevented (DPPs). Clearly, as the life years gained are calculated as weights for each DPP, assumptions which weaken the DPP model will, therefore, impact upon the results of the LYG model as well. However, it was not our purpose to evaluate the specific results of either model but rather to assess the use of time-based outcome (LYG) versus an event-based outcome (DPP). Another limitation of our study is that we have not quantified the uncertainty around each assumption in this paper and this may be warranted in future efforts in order to identify where to focus further detailed work and clarifications.

Lastly, here we have considered just two metrics for capturing changes in disease occurrence. Our argument generalises to choice from among the full spectrum of available metrics - from the purely objective metrics of aetiologic epidemiology, to metrics based in objective phenomena but weighted to take account of social values (such as life years lost and gained), to hybrid measures such as the DALY which entail some formal valuing of health states but which maintain strong links to the 'objectivist' categories of medical science, to metrics located within the subjectivist theory of value of economics ('extra-welfarist' QALYs and 'welfarist' QALYs [21]). Methodological pluralism can enrich public health. No one metric meets all purposes, so choices from among them should be deliberate and justified.

## Conclusion

In conclusion, time-based metrics (such as life years) are generally preferable to simply enumerating deaths prevented, because life years direct attention to the changes in the natural history of disease that are produced by changes in key health determinants. The life-years metric also better reflects social preferences.

## Competing interests

The authors declare that they have no competing interests.

## Authors' contributions

HG conducted the analysis and drafted the manuscript, JC helped with the analysis and edited the manuscript, JP contributed to the design of the study, helped with the analysis and with drafting the manuscript. JP and SC conceived of the study. All authors read and approved the final manuscript.

## Pre-publication history

The pre-publication history for this paper can be accessed here:

http://www.biomedcentral.com/1471-2458/12/88/prepub
